# Temporomandibular joint ankylosis in a child: an unusual case with delayed surgical intervention

**DOI:** 10.1186/s12887-015-0495-4

**Published:** 2015-11-06

**Authors:** Ching Ching Yew, Shaifulizan Ab Rahman, Mohammad Khursheed Alam

**Affiliations:** School of Dental Sciences, University Science Malaysia, Health Campus, 16150 Kubang Kerian, Kelantan Malaysia; Oral and Maxillofacial Surgery Department, School of Dental Sciences, University Science Malaysia, Health Campus, 16150 Kubang Kerian, Kelantan Malaysia

**Keywords:** Temporomandibular joint ankylosis, Septic arthritis, Interpositional arthroplasty, Timing for surgery

## Abstract

**Background:**

The Temporomandibular Joint (TMJ) ankylosis in child is rare and yet the causes still remain unclear. This condition that affects the feeding and possible airway obstruction do not only worry the parents, but also possesses as a great challenge to the surgeons. Furthermore, it interferes with the facial skeletal and dento-alveolar development in the on growing child.

**Case presentation:**

In this case report, we presented the management of a 7 year old with left TMJ ankylosis discovered since infant. Clinical and imaging investigations were consistent with left temporomandibular joint ankylosis (Type IV) possible secondary to childhood septic arthritis. Left gap arthroplasty via modified Al Kayat Bramley and retromandibular approach was performed, with interpositional arthroplasty placement of temporalis fascia graft. No complications from the surgery except reduced mouth opening were seen. Possible contributing factors to this less than satisfactory mouth opening are adressed.

**Conclusion:**

We describe here, an unusual childhood temporomandibular joint ankylosis possible due to septic arthritis with delayed surgical intervention. The aetiology, classifications, timing and choice of surgical techniques along with its considerations and complications are discussed. Although there is no consensus on the surgical treatment of TMJ ankylosis, early mobilisation, aggressive physiotherapy and close follow-up are advocated by many authors for successful treatment.

## Background

Temporomandibular joint (TMJ) ankylosis is a joint disorder which refers to bone or fibrous adhesion of the anatomic joint components, resulting in loss of function [1].

The etiologies of TMJ ankylosis include previous trauma, previous TMJ surgery, arthritis, and infection. It can be congenital, and in some cases, idiopathic. The most common etiology of TMJ ankylosis is previous trauma, with the second being infection [2–4].

Problems associated with TMJ ankylosis in a child include issues with airway maintenance, feeding difficulties and speech development alterations. Furthermore, it interferes with the facial skeletal and dento-alveolar development in the on growing child. Severe facial disfigurement can aggravates psychological stress and further decreases the patient’s quality of life [5].

Hence, timely diagnosis of TMJ ankylosis, especially in children, and early surgical intervention must be applied to prevent growth alterations [6].

We present a case of a 7 year old child with left TMJ ankylosis discovered since infant, as well as the discussion and complications of the surgery.

## Case presentation

**Fig. 1:**
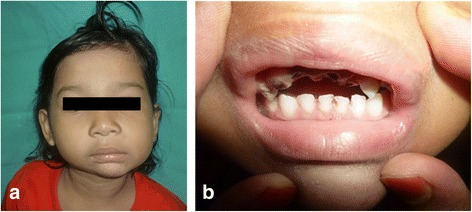
**a** and **b**: Patient presentation at age 3, extraorally and intraorally

**Fig. 2:**
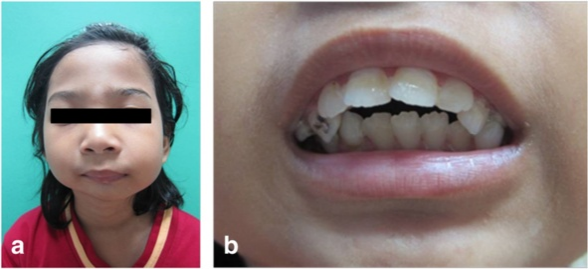
**a** and **b**: Patient presentation at age 7, extraorally and intraorally

A 3 year’s old Malay girl from Terengganu was first presented to the Department of Oral and Maxillofacial Surgery, Hospital University Science Malaysia in year 2009. She came with her mother, who noticed limited mouth opening of the child since 1 year old. The limited mouth opening did not interfere with feeding, but she claimed it was difficult for the child to perform proper tooth brushing (Figs. 1 and 2).

Relevant past medical history revealed that she was born following an uncomplicated pregnancy via spontaneous vaginal delivery, and she was not a syndromic child. She was admitted though, at day 8 life, for bronchopneumonia and left knee septic arthritis. Blood culture and sensitivity indicated presence of *Staphylococcus aureus* infection, following the child was treated with cloxacillin and subsequently discharged.

She was the second youngest child from ten siblings, and comes from a poor social economic background where both parents were primary school teachers. Unfortunately, she was lost to follow up when her father suffered from cerebral vascular accident in year 2010 and was hemiplegic ever since. Being the sole bread winner and on top of the overwhelming parenthood responsibilities, the apprehensive mother had prevented the girl from receiving any early surgical interventions. The patient finally returned to our clinic at the age of 7, accompanied by the mother whom noticed a significant delay in speech development in her.

General examination showed she has normal growth spurt, with average height and weight in comparison with the local Malay population. Her cognitive development is up to par.

On extra-oral examination, the child presented with asymmetrical face, with reduced lower facial height, and deviated chin point to the left side. She also possessed a relatively small mandible, with a convex side profile. No movement of the left temporomandibular joint (TMJ) can be palpated via the external auditory canal. No mouth opening can be observed at all.

Surprisingly, patient presented with excellent oral hygiene intraorally. There is incomplete bite, and slight increase in the upper incisor proclination.

**Fig. 3:**
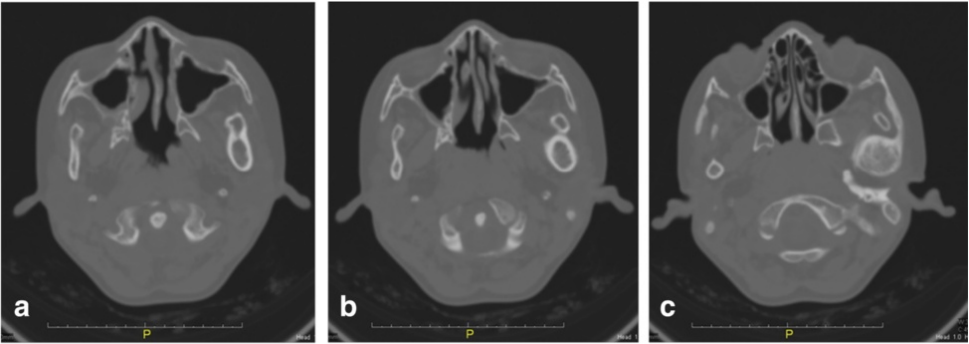
**a**, **b** and **c**: Axial views of CT scan, showing the bony expansion medio-lateraly from the left ascending ramus to the condylar head

**Fig. 4:**
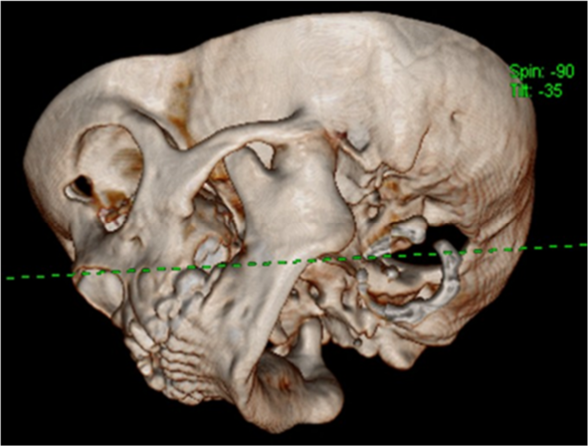
3D reconstruction of left TMJ showing the fusion of the left condylar head to the base of skull

Further imaging with CT scan showed that there is bony fusion between the left condylar head and the base of the skull, with evident of sclerosis and enlargement of the condylar head, extending into the sigmoid notch. The left TMJ space was entirely obliterated with bony deposition (Fig. 3a, b, c). Three-dimensional reconstruction of CT imaging confirmed the fusion of the left TMJ, and depicted the shortening of the antero-posterior dimension of the mandible in the ankylotic left side as compared to the normal right side of mandible (Fig. 4).

The child was diagnosed with left temporomandibular joint ankylosis (Type IV) possible secondary to childhood infection. Left gap arthroplasty via modified Al Kayat Bramley and retromandibular approach was performed, with interpositional arthroplasty placement of temporalis fascia graft.

**Fig. 5:**
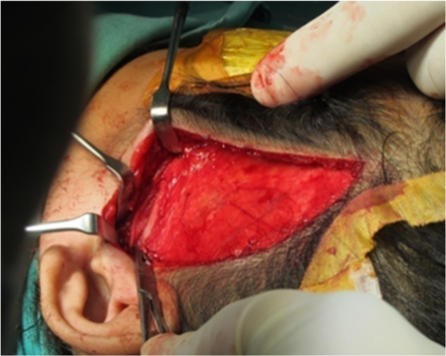
Modified Al Kayat Bramley flap was raised up to the zygomatic arch

**Fig. 6:**
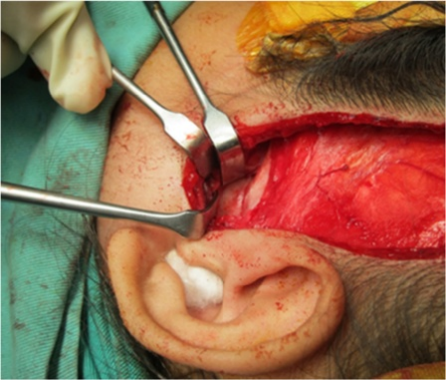
Bony fusion of left TMJ

The surgery was performed under general anaesthesia with nasotracheal intubation assisted by fibre optic scope. Exposure of the left temporal region was done and an extended periauricular incision was made and deepened to the superficial temporalis fascia by using a combination of blunt and sharp dissection. The flap was raised up to the zygomatic arch where the periosteum was incised on the most posterior aspect of the zygomatic arch (Fig. 5). The subperiosteal plane of dissection was performed until the capsule of the joint was visible, followed by a T shape incision to expose the joint space and bony ankylosis. Dense, hard sclerotic bone was observed around the left TMJ (Fig. 6). To prevent injury to the zygomatic branch of facial nerve, the exposure was not extended more inferiorly; instead the retromandibular approach was performed in adjunct to help identify the distorted anatomy around the left TMJ.

**Fig. 7:**
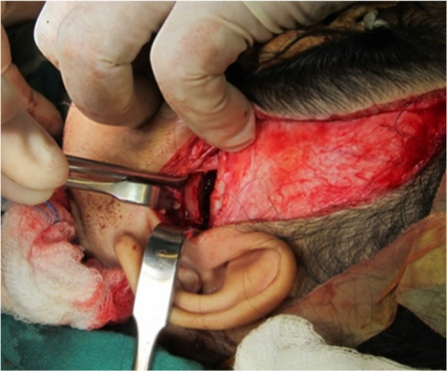
Gap arthroplasty of left TMJ

**Fig. 8:**
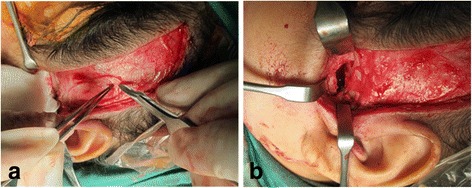
**a** and **b**: Temporal fascia graft was harvested and rotated above the zygomatic arch, into the temporomandibular joint

Ankylotic bone mass was removed using a fissure bur until a thin layer of bone remained on the most medial aspect of the bony union. To prevent injuries to the internal maxillary artery or pterygoid plexus of veins, the osteotomy was completed very carefully with a chisel. The condylar stump and glenoid fossa were recontoured with surgical shaving burs. Intra-operatively, a gap of 15 mm in the left TMJ was created (Fig. 7) and maximum interincisal opening of 25 mm was recorded. The temporal fascia graft was harvested according to the size of the defect, and rotated above the zygomatic arch, into the temporomandibular joint as the interpositional tissue and secured with sutures (Fig. 8).

**Fig. 9:**
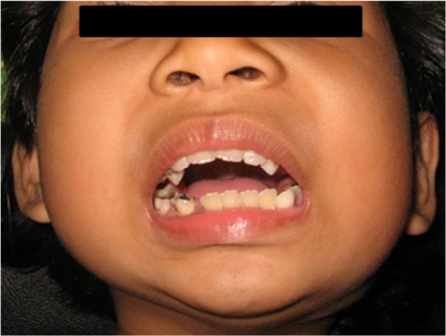
3 month post-operative mouth opening

Post-operatively, all surgical wounds healed uneventfully, and patient showed no signs of facial paresis or other complications such as anterior open bite and Frey’s syndrome. She was placed under strict physiotherapy exercise commencing one week post operatively; however the child did not adhere to the physical therapy under parental supervision. Although patient could not attend to the hospital for periodical follow ups due to socio-economy and logistical constraints, home visits were extended and revealed that her maximum interincisal opening had reduced to 20 mm at 3 month post-operatively (Fig. 9). Further telephone interviews confirmed that the mouth opening had remained the same till date (15 months post-operatively). She is encouraged to continue with vigorous mouth opening exercise using wooden spatulas and is still under review.

## Discussion

**Fig. 10:**
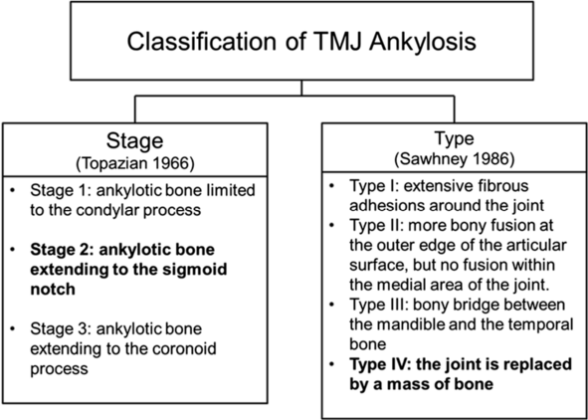
Classification of TMJ ankylosis

The severity of TMJ ankylosis can be classified by location, stage/extent or type of tissue involved [7, 8] (Fig. 10).

This case report featured a stage 2 ankylosis, where the extension of the lesion had involved the sigmoid notch. It is a type IV classification where the joint is totally obliterated by an expanded bony block between the ramus and the skull.

In complete ankylosis, the maximum inter-incisal opening is ≤5 mm, whereas it is ≥5 mm in incomplete ankylosis [9]. The child in this case was presented with complete ankylosis, where no mouth opening was observed at all.

TMJ ankylosis is caused by a variety of conditions such as local or systemic infections, TMJ arthritis, trauma, and neoplasm. The most common etiology of TMJ ankylosis is previous trauma, with the second being infection [3, 10]. TMJ ankylosis as a complication of infection is a known but extremely rare condition, with only few reported scientific literature. TMJ ankylosis associated with undiagnosed septic arthritis may not be diagnosed until many years later [11, 12]. Septic arthritis infections with involvement of the TMJ region that could lead to ankylosis are often caused by *Staphylococus aureus* [13].

Although it cannot be proven, we feel that this was most likely a rare complication of neonatal bronchopneumonia and left knee septic arthritis that may have spread hematogenously to the left TMJ.

TMJ ankylosis should be treated as soon as the condition is diagnosed. In children especially, the aim of early treatment is to restore mandibular mobility and to enhance further growth to reduce the possibility of future facial asymmetry. The short ramus condyle unit can result in subsequent emergence of unilateral mandibular retrusion, significant malocclusion, and can restrict mid-facial growth [14].

Therefore, treatment of ankylosis should commence as soon as patient’s co-operation after the operation is expected. The surgical team must take time to explain to the child, in an age appropriate way, the operation and post-operative physical therapy program. The parents must also be included as active participants in the overall management. After evaluation, the operation might be delayed if appears unlikely the patient and family can manage with the procedure, such as in this case. Kaban suggested children of 3 years of age and older are suitable candidates for ankylosis release. It is not necessary to wait for growth completion when deciding the timing of surgery [15].

Surgeons must also bear in mind that manipulation of mandible is difficult in infants because of the soft alveolar ridge and the fragile mandible bone, and excessive force may lead to jaw fracture that can further complicate airway management. Also, surgical intervention in early life will subject patient to high risk of injury to the crucial anatomical structures, such as the facial nerve and maxillary artery [5].

After taking all factors into considerations, the earliest possible age of 7 was decided in this case for surgical intervention. This is a decision made not only by the surgical team, but also after careful considerations on the family’s priority.

Surgical intervention is the only logical solution to overcome this problem. The aim of the surgical treatment is to remove the ankylotic part, re-establish the joint function and to prevent re-ankylosis [15].

Many surgical techniques have been described for the treatment of TMJ ankylosis, but there is no agreed treatment till date, and results have been variable and often less than satisfatory [16, 17]. The surgical procedures can be classified into 4 groups as shown (Table 1).

**Table 1:** Different surgical procedures in treating TMJ ankylosis

Procedure	Description
Gap arthroplasty [20]	This is the oldest surgical method and consists of resection of the bone only without any interpositional tissue or material.
Interpositional arthroplasty with autogenous grafts [7]	This involves gap arthroplasty and interpositional of autogenous tissues such as contochondrol grafts, temporalis muscle flaps, dermal grafts, auricular cartilage and fascia.
Interpositional arthroplasty with alloplastic materials [8, 21]	This involves the use of lyophilized dura mater or alloplastic materials such as vitalium, acylic, Teflon-proplast and silicone.
Placement of the hemijoint or total prosthesis [22]	Usually indicated for failed multioperated cases, example in advanced degenerative osteoarthritis or oncology cases.

Gap arthroplasty is a simple method with short operating time. However, this technique is reported with high rate of recurrence [3]. Besides, gap arthroplasty without interposition requires a large amount of bone resection. Mouth deviation is the result of this operation.

Hence, surgical technique interpositional arthroplasty with temporalis fascia was chosen in this case. The temporalis flap is the most widely used interposition materials in OMF region. It has the advantage of being an autogenous material, has the donor site in the surgical field and is easy to prepare. This flap could mimic the physiologic function of the disc and works as barrier to bony ankylosis [18]. The temporalis fascia is less bulky than the temporalis muscle flap when it was rotated over the zygomatic arch and has aesthetic advantages [19].

Based on current literatures, the ideal treatment option of costochondral graft reconstruction following interpositional arthroplasty is indicated in children, especially in such case of significant facial deformity [4]. However, lack of parental acceptance and consent on harvesting a rib graft had precluded that option.

The most common complications after ankylosis surgery are limited mouth opening and reankylosis. Temporary paresis of facial nerve, anterior open bite and Frey’s syndrome has also been encountered [1].

In this case, the only complication noted was limited mouth opening. Erol et al. recorded an average maximum interincisal opening of 30.7 mm in their clinical study, which uses technique of interpositional arthroplasty with temporal fascia and muscle flap. The decision of not proceeding with coronoidectomy intra-operatively was made based on absence of visible coronoid elongation clinically and radiographically. However, in view of the less than ideal mouth opening post-operatively, the authors agreed that coronoidectomy could have been a wiser decision and is perusing the parents to consent for it.

The authors wish to highlight a few possibilities that could lead to the current limited mouth opening: First, failure to address the ipsilateral and/or contralateral coronoid process; Second, undissected tendon of the masseter and medial pterygoid muscles [4]; and Third, lack of compliance to post-operative aggressive physical therapy. The already formidable challenge of managing TMJ ankylosis among children can be further complicated if unmet with appropriate parental acceptance and compliance in the perioperative management. All these limitations can serve as learning issues in the management of future cases by all readers.

Although in this case, patient’s mouth maximum interincisal opening was only 20 mm and is less than ideal, it is still acceptable in relation to her severely retruded and underdeveloped mandible. She was able to consume normal diet and perform oral hygiene measure such as tooth brushing. Her current 15 months post-operative maximum interincisal opening had remained at 20 mm currently and is still under review.

Future planning for this patient includes coronoidectomy to improve her mouth opening, followed by distraction osteogenesis, and possible orthonagthic surgery later in her young adulthood to correct any remaining dental and skeletal deformities. The option of costochondral graft unfortunately is not well accepted by the parents and can only be reconsidered when the child reaches the age of consent.

## Conclusion

Regardless of the aetiology of TMJ ankylosis, early surgical intervention is indicated to facilitate feeding, speech, and maxillofacial growth development.

However, risk of injury to the nearby anatomical structures and commitment from the patient and family must be taken into consideration when deciding the timing of surgery.

Although there is no consensus on the surgical treatment of TMJ ankylosis, early mobilisation, aggressive physiotherapy and close follow-up are advocated by many authors for successful treatment. It is not an understatement that surgical intervention itself do not guarantee success of the treatment, as post-operative physical therapy is equally, if not more important, to maintain mouth opening and to prevent reankylosis of the TMJ.

## Consent

Written informed consent was obtained from the patient’s mother for publication of this Case report and any accompanying images. A copy of the written consent is available for review by the Editor of this journal.

## Abbreviations

TMJ: Temporomandibular Joint
